# BOWENOID PAPULOSIS OF THE GENITALIA SUCCESSFULLY TREATED WITH TOPICAL TAZAROTENE: A REPORT OF TWO CASES

**DOI:** 10.4103/0019-5154.55643

**Published:** 2009

**Authors:** Veeranna Shastry, Jayadev Betkerur, Kushalappa

**Affiliations:** *From the Department of Skin and STD, J.S.S Hospital, Mysore, Karnataka, India.*

**Keywords:** *Bowenoid papulosis*, *human papilloma virus*, *tazarotene*

## Abstract

Bowenoid papulosis is a rare condition of the genital area caused by human papilloma virus. Clinically, it resembles viral wart and histopathologically Bowen's disease. We herein report two male patients presenting with multiple flat papules on the penis and scrotum. The second patient was HIV-positive. Histopathology showed features of bowenoid papulosis. Both the patients were treated with topical tazarotene gel 0.05%. Lesions cleared within 2 weeks in both the patients.Second patient had recurrences that cleared after retreatment with tazarotene.

## Introduction

Bowenoid papulosis (BP) is a distinct clinicopathologic entity characterized by multiple, small skin colored to reddish brown papules, primarily occurring on the genitalia of young adults. BP is strongly associated with human papilloma virus (HPV) infection and is difficult to differentiate clinically and histopathologically from squamous cell carcinoma *in situ*.[1] It is often considered as low grade *in situ* carcinoma.[2]

We herein report two cases of BP of genitalia successfully treated with topical tazarotene.

## Case Reports

### Case 1

**Figure 1:**
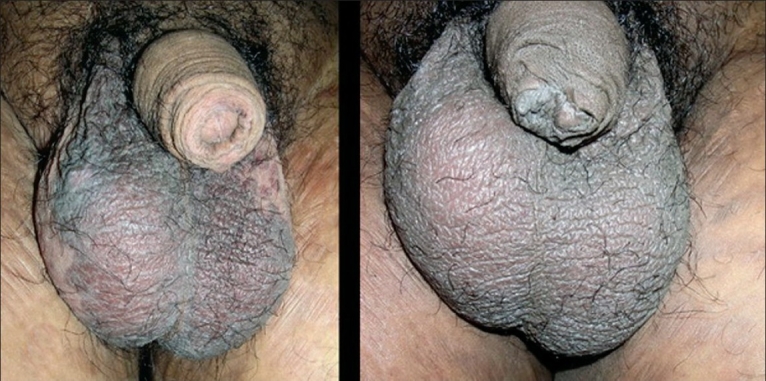
Case one - Lesion on the scrotum (a) before treatment and (b) after two weeks of treatment

**Figure 2:**
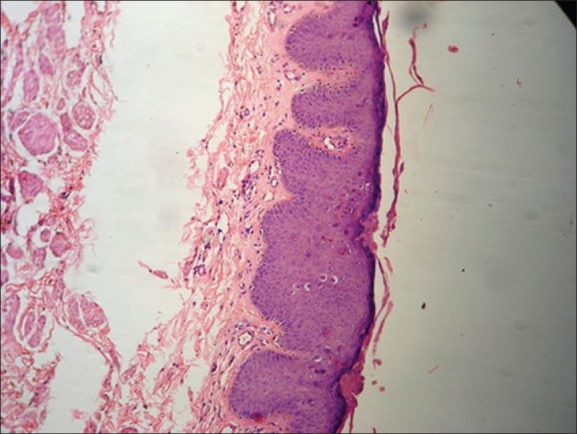
Case one - histopathology showing acanthosis and dyskeratosis H and E, ×10

**Figure 3:**
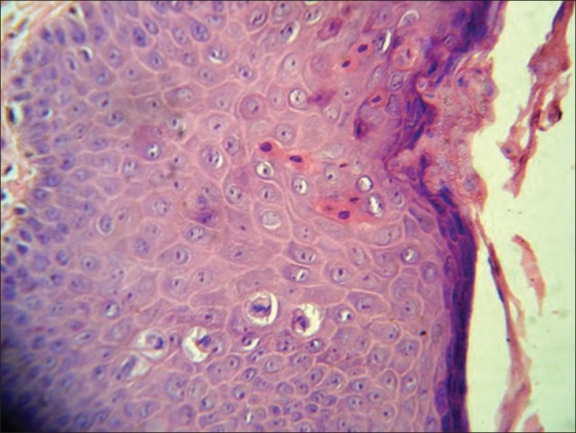
Case one - histopathology showing epidermal dysmaturation and dyskeratosis, H and E, ×45

A 31-year-old, unmarried male presented with asymptomatic lesions over the scrotum for the past one year. Earlier treatment with topical steroids and antifungal agents had not shown any improvement. On examination, there was a large, well-defined, pigmented plaque with velvety surface over the scrotum [Figure 1a]. There were similar smaller papules present on the adjacent area and over the prepuce. Blood VDRL and HIV screening test were nonreactive. Skin biopsy from the lesion over the scrotum showed irregular acanthosis, disordered maturation of the epidermis, crowding of the nuclei, focal hypergranulosis, dyskeratosis, hyperchromatic, and multinucleated keratinocytes with increased mitosis [Figures 2 and 3].

On the basis of the clinical and histological features, the case was diagnosed as bowenoid papulosis and the patient was treated with tazarotene gel 0.05% once daily. After one week, lesion became erythematous and scaly with mild irritation and the treatment was stopped. The lesions completely cleared in the next week [Figure 1b]. The patient is under follow up for one year and there is no relapse.

### Case 2

**Figure 4:**
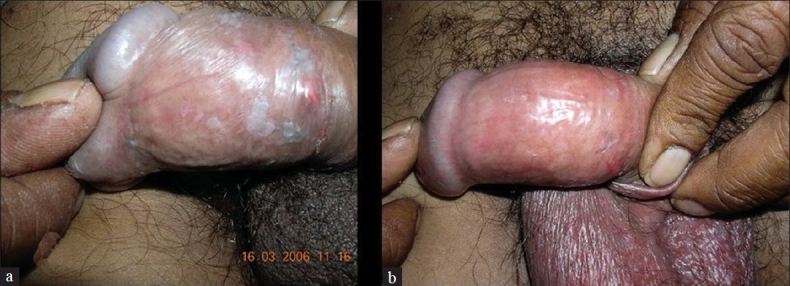
Case two - Lesion on the penis (a) before treatment and (b) after two weeks of treatment

**Figure 5:**
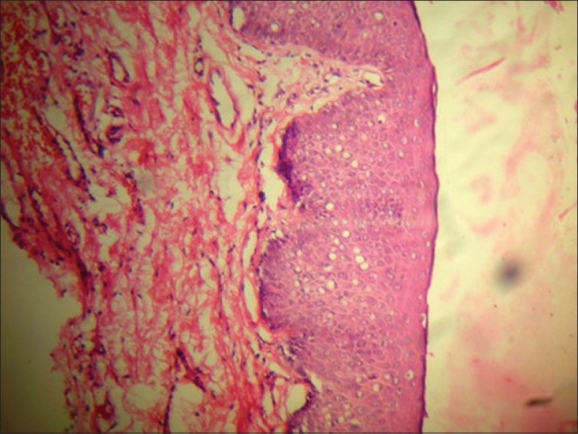
Case two - histopathology showing acanthosis and epidermal dysmaturation, H and E, ×10

**Figure 6:**
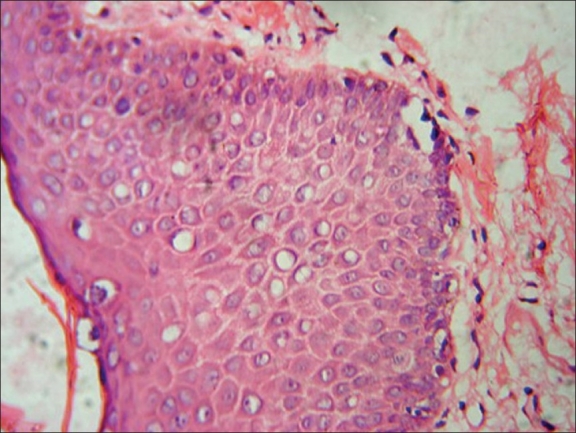
Case two - histopathology showing epidermal dysmaturation, H and E, ×45

A 40-year-old male presented with asymptomatic lesions of three months duration over the penis. Previous treatment with topical antibiotics and antifungal agents failed to clear the lesions. Examination revealed multiple, well-defined, flat pigmented papules with velvety surface [Figure 4a]. The lesions were situated over the prepuce. Hemogram and urine analysis was within normal limits. ELISA test for HIV antibodies was positive. Skin biopsy from the lesion showed irregular acanthosis, vacuolated cells, pleomorphic cells, and dysmaturation [Figures 5 and 6].

The patient was treated with topical tazarotene 0.05% once daily. Although he had mild irritation initially, he continued the treatment and the lesions resolved within two weeks [Figure 2b]. After one month, few fresh lesions reappeared, which were again cleared with tazarotene.

## Discussion

Bowenoid papulosis was first described by Kopf and Bart in 1977.[2] Some authors prefer to use the term penile intraepithelial neoplasia, but there is no formal consensus on the clinicopathological classification. An alternative expression of squamous intraepithelial lesion (SIL) has been proposed.[3] BP is probably a virus-induced epithelial dysplasia mainly associated with Human papilloma virus type 16 but other types have also been found.[3] HIV- infected individuals are at increased risk for persistent HPV infection and HPV-associated anogenital SIL.[1] It usually affects sexually active adults with slight female predominance and is more common in smokers. It presents as asymptomatic, flat, hyperpigmented, or violaceous papules, few millimeters to several centimeters in size, occurring over the penis, vulva, or perianally.[2] Lesions are also reported in the oral cavity and lower abdomen.[4]

Histopathology shows features resembling Bowen's disease. There is crowding and an irregular windblown arrangement of nuclei, many of which are large, hyper chromatic, and pleomorphic. Dyskeratosis atypical mitosis and multinucleated keratinocytes are also present.[5]

Over time, these lesions can regress, persists, recur, or progress to invasive squamous cell carcinoma in some cases. Female partners of men with BP and women with BP have a risk of cervical dysplasia. The condition has to be differentiated from genital warts, melanocytic nevi, seborrheic keratosis, lichen planus, Zoon's balanitis, squamous cell carcinoma, basal cell carcinoma, and anogenital Bowen's disease.[2]

Treatment of this condition is mainly by local agents such as 5 fluorouracil, imiquimod, podophylin, and cidofovir. Other modalities include excision, electrocautery, CO_2_ laser, cryosurgery, photodynamic therapy, and Interferon.[2,3] Retinoid either topically or systemically is also effective in treating this condition.[6,7] Therapeutic vaccination has also been tried.[8]

Tazarotene is a polyaromatic retinoid that influences epithelial cell proliferation and differentiation. It is mainly indicated in the management of acne vulgaris and psoriasis. It is also found to be useful in congenital icthyosis,[9] icthyosis bullosa of siemens,[10] nevus comedonicus,[11] linear Darier's disease,[12] elephantiasis verrucosa nostra,[13] basal cell carcinoma,[14] and *in situ* squamous cell carcinoma.[15]

Both of our patients had clinical and histological features of BP. The lesions completely regressed in both the patients after treatment with tazarotene. Mild burning sensation and erythema at the site of application were the only adverse effects observed in our cases. The rapid clinical response was more likely to be due to the effect of the drug rather than due to natural course of disease. Absence of recurrence in first case and successful retreatment in the second case also support the effectiveness of topical tazarotene in BP.

BP being asymptomatic often goes unnoticed. Though this condition is considered as a low grade *in situ* carcinoma, the prognosis is good in majority of the patients. Many drugs have been used to treat BP. Topical tazarotene has never been tried in this condition to the best of our knowledge. Our results suggest that topical tazarotene is an effective treatment for BP. It also offers faster and longer remissions. However, multicentric studies on a large number of patients are warranted to substantiate our results.
